# Supplementary material for: Gender differences in factors influencing intention to undergo cardiovascular disease health checks: A cross-sectional survey
Source: PLoS One. 2020 Sep 24;15(9):e0239679. doi: 10.1371/journal.pone.0239679 (PMC7514016; doi:10.1371/journal.pone.0239679)
Supplement: S1 Table — (PDF) [file pone.0239679.s002.pdf]

Table 1 Summary results of presumption checked

Gender	Outcome	Determinants	Presumptions checked: Deviance value	df	Value/df	Akaike's information criterion (AIC)	Test of parallel lines (p-value)	Pseudo R-square (Nagelkerke)	VIF for the independent variables
Men	Degree of likeliness to undergo CVD health checks	First block: internal factors	271.043	311	0.872	289.043	0.047	31.6%	1.103-2.166
		Second block: internal and external factors	267.128	309	0.864	289.128	0.068	32.8%	1.321-2.331
		Third block: internal and external factors with control of demographics, morbidities and history of regular health check	224.798	298	0.754	268.798	0.002	61.6%	1.073-2.459
	Likely timeline of the public to undergo CVD health checks	First block: internal factors	394.214	470	0.839	414.214	0.093	9.6%	1.103-2.166
		Second block: internal and external factors	391.151	468	0.836	415.151	0.348	11.4%	1.321-2.331
		Third block: internal and external factors with control of demographics, morbidities and history of regular health check	363.649	457	0.796	409.649	0.135	34.5%	1.073-2.459
Women	Degree of likeliness to undergo CVD health checks	First block: internal factors	452.497	465	0.973	470.497	0.019	18.9%	1.065-1.635
		Second block: internal and external factors	441.841	463	0.954	463.841	0.023	22.0%	1.072-1.687
		Third block: internal and external factors with control of demographics, morbidities and history of regular health check	379.814	451	0.842	425.814	0.161	46.0%	1.085-1.728
	Likely timeline of the public to undergo CVD health checks	First block: internal factors	580.600	701	0.828	600.600	0.015	22.1%	1.065-1.635
		Second block: internal and external factors	568.692	699	0.814	592.692	0.051	26.3%	1.072-1.687
		Third block: internal and external factors with control of demographics, morbidities and history of regular health check	524.238	687	0.763	572.238	1.000	41.8%	1.085-1.728
